# Free Time For Wellness: a co-designed intervention utilizing social networks to encourage physical activity for cancer prevention among low resourced mothers

**DOI:** 10.1186/s12889-021-11775-9

**Published:** 2021-10-07

**Authors:** Lauren C. Houghton, Marley P. Gibbons, Jeanette Shekelle, Ingrid Oakley-Girvan, Jessica L. Watterson, Kate Magsamen-Conrad, Cheryl Jones, Kajal Gokal

**Affiliations:** 1 Department of Epidemiology, Columbia University Mailman School of Public Health, 722 W 168th Street, New York, NY 10032 USA; 2 Herbert Irving Comprehensive Cancer Center, 1130 St Nicholas Avenue, New York, NY 10032 USA; 3 The Public Health Institute, The Data and Technology Proving Ground Program, 555 12th Ave, 10th Floor, Oakland, CA 94607 USA; 4 Medable Inc, 525 University Ave, Ste A70, Palo Alto, CA 94301 USA; 5 Jeffrey Cheah School of Medicine and Health Sciences, Monash University Malaysia, Subang Jaya, Malaysia; 6 Center for Healthcare Organizational and Innovation Research (CHOIR), School of Public Health, University of California, Berkeley, 50 University Hall, Berkeley, CA 94704 USA; 7 Department of Communication Studies, The University of Iowa, 257 Becker Communication Studies Building, Iowa City, IA 52245 USA; 8 Holden Comprehensive Care Center, 200 Hawkins Drive, Iowa City, IA 52242 USA; 9 Manchester Centre for Health Economics, The University of Manchester, Oxford Road, Manchester, M13 9PL UK; 10 National Centre for Sport and Exercise Medicine (NCSEM), School of Sport, Exercise and Health Sciences, Loughborough University, Leicestershire, LE11 3TU UK; 11 The Centre for Lifestyle Medicine and Behaviour, School of Sport, Exercise and Health Sciences, Loughborough University, LE11 3TU Leicestershire, UK

**Keywords:** Community, Co-design, Population health, cancer prevention, Technology, Wellness

## Abstract

**Background:**

Physical activity is central to chronic disease prevention. Low resource mothers face structural barriers preventing them from increasing their physical activity to reduce their chronic disease risk. We co-designed an intervention, with the ultimate goal of building social cohesion through social media to increase physical activity for low resourced mothers in urban settings.

**Methods:**

In 2019, we interviewed 10 mothers of children (< 12 years) living in Washington Heights, Manhattan. The interviews were transcribed and coded for themes that guided the creation of a co-design workshop. Washington Heights-based mothers (*n* = 16) attended a co-design workshop to generate the blueprint for the Free Time for Wellness intervention.

**Results:**

Mothers in our sample had limited time, external support and resources, which hindered them from increasing their physical activity; we learned that in addition to physical health, mental health was a concern for participants. Participants had varying degrees of self-efficacy and trust in social media. Bringing mothers and researchers together in a co-design workshop, we identified types of physical activities they would enjoy participating in, the ideal time to do so, the kind of childcare they needed, and their preferences for communication with the community champion. The interviews and workshop highlighted the need for a community space that mothers and children could co-occupy. The intervention was designed to be 3 months’ worth of sample programming with one activity per week, rotating between dance, yoga, food pantry visits and group playdates. Participants were invited to bring their children to a space with one room for the ‘participants only’ activity and a second room in which professional childcare providers supervised the children.

**Conclusions:**

Through this two-phased co-design process, we created an intervention with mothers in an urban community with the goal of using social media to bring them together for wellness, primarily through increased physical activity. Despite the co-design of this intervention with a specific community, there are some universal applications of our findings, and of the use of co-design workshops, to other settings.

**Supplementary Information:**

The online version contains supplementary material available at 10.1186/s12889-021-11775-9.

## Background

Over 50% of U.S. men and women are failing to reach the recommended guidelines of 150 min of moderate to vigorous physical activity each week [1]. Physical inactivity is particularly prevalent in women with low socioeconomic position (60% inactive) [1], suggesting that there are structural barriers to being physically active. This is because, as McNeil et al. (2006) explain, the social environment in which individuals live is important in shaping physical activity and health outcomes [2]. Communities with high levels of social inequality are at increased risk of developing conditions like cancer, heart disease, diabetes, and other chronic diseases. One aspect of social inequality, socioeconomic position is positively associated with physical activity. Proposed mechanisms through which socioeconomic position may influence physical activity include biological stress, access to healthcare and other resources like recreational facilities and material resources to access those facilities, such as gym memberships [2]. Therefore, this study will focus on low resourced mothers within a community of high social inequality, where the risk of chronic disease is higher and there is greater potential to make an impact on these health disparities.

While interventions aimed at increasing physical activity can reduce the burden of chronic disease among the socially disadvantaged, most physical activity interventions are aimed at the individual-level rather than the community-level [3]. Exciting and expanding research on social networks and social cohesion suggests that individual adoption of health behavior is much more likely when participants receive social reinforcement from multiple neighbors in their social network [4–6] and is facilitated by a representative in the community (community champion) [7]. Social reinforcement is an aspect of a cohesive society which works towards the well-being of all its members, fights exclusion and marginalization, creates a sense of belonging, promotes trust, and offers its members the opportunity of upward social mobility [8–10]. Increasing resources by building social cohesion is a stealth approach that intervenes on structural barriers to increase physical activity in at-risk populations.

Low resourced mothers are a particularly vulnerable, yet simultaneously a powerful group. They are particularly at risk for chronic diseases, such as cancer [11], and increased burden to improve critical health behaviors, due to challenges such as limited time, resources, and access to safe neighbourhoods [12]. In terms of breast cancer, mothers are at a particular increased risk for 10 years after childbirth [13]. At the same time, mothers make decisions that affect family health and model behavior for their children. Therefore, if interventions can change mothers’ physical activity, they may also impact their children’s health [14]. We have demonstrated such intergenerational impact previously with regard to maternal weight gain during pregnancy and daughters’ body size throughout life [15]. Furthermore, there is the existing cultural practice of mothers joining mother groups and subscribing to “mommy blogs” to help guide and support each other while raising children [16]. The saying “it takes a village” captures this cultural phenomenon, and it is this cultural context, coupled with the science of social networks and co-design methodology, that we integrated for this study, Free Time for Wellness (FT4W).

This project and our study team is the result of a National Cancer Institute (NCI)/ Cancer Research UK (CRUK) Sandpit workshop focused on developing innovative interventions for cancer prevention using technology. During this 4-day workshop, the multi-disciplinary research team devised the overall goal to develop an innovative intervention; to build social cohesion among a community of mothers through neighborhood-based mobile applications. Our initial idea was to co-design an intervention to build social cohesion in order to create free time for mothers who lacked resources to pay for conveniences such as childcare, food preparation, and house cleaning responsibilities. These duties are disproportionately put on mothers and can be time intensive. Our initial ideas are described in more detail elsewhere [17] and include linking mothers through NextDoor – a neighborhood hub and social networking service (application and website) providing verified connections between neighbors and the exchange of information, goods and services—to find alternative childcare and to organize group wellness activities. We planned to include community champions to facilitate social cohesion and nudge the use of FT4W. Recognizing our privileged positionality and biases in relation to this topic, we chose to engage the target population to explore their health concerns and inform the development of the intervention.

The overall objective of this two phased study was to explore the needs and preferences of low resourced mothers and design an innovative lifestyle intervention using technology to encourage physical activity and ultimately reduce the risk of chronic diseases, such as cancer.

We did this through two aims:

1. identify barriers and facilitators of free time for physical activity among mothers from a low-resource population through qualitative semi-structured interviews
2. co-design an innovative lifestyle intervention *for* mothers *with* mothers who are most likely to use it.

## Methods

### Study population

The study was conducted in the Washington Heights neighborhood of Manhattan. While the neighborhood is heavily LatinX (70%), its population reflects the diversity of New York City as a whole with 7.7 and 17.7% of the remaining residents being African American and White, respectively [18]. The area was selected for its urban, low income setting, as well as our team’s established community connections.

We partnered with Columbia University’s Mailman School of Public Health’s Washington Heights-based community space, the Columbia Community Partnership for Health (CCPH) due to its strong history of creating and supporting community wellness through the Community Engagement Core Resource (CECR) [19] for recruitment and use of the space.

### Aim 1: semi-structured interviews to identify barriers and facilitators of free time for physical activity

#### Recruitment

We worked with CCPH to recruit a convenience sample of participants for the semi-structured interviews.

The eligibility criteria specified mothers with children under 12 years of age, residents of Washington Heights, and smart phone users. Our rationale for including mothers of children less than age 12 was because younger children require more care and thus time, and, we wanted to intervene with cancer prevention in premenopausal women. The neighborhood-based app ‘NextDoor’ was not available in Spanish, limiting our sample to those that spoke conversational English or were bilingual. CCPH advertised research volunteer opportunities during community outreach events on their digital signage via newsletters and ad hoc emails to their list-serve of community residents, and on their community health websites, GetHealthyHeights.org and GetHealthyHarlem.org. Mothers interested in participating expressed their interest to CCPH and provided a contact telephone number. The study’s project coordinator (MG) contacted potential participants by phone to confirm they met the eligibility requirements, and, if so, then scheduled an intake interview. We recruited additional participants through a digital flyer that was posted on local Facebook groups and ‘mom list serves’- email mailing lists for subscribing mothers, usually organized by neighborhood.

#### Semi-structured interviews

We (LCH and MG) conducted semi-structured interviews in person, at CCPH between May 23 and June 17, 2019. We planned to interview a sample of 12 women or until saturation was met using an interview guide covering four general themes (time & time management, health, technology & smartphones and resources; see supplemental materials). A professional transcription company transcribed audio recordings of the interviews. The research team then uploaded the transcriptions into Dedoose software (version 8.2.31).

#### Analysis

One researcher (JS) who was blinded to the research questions, coded all transcripts openly, allowing themes to arise inductively, following grounded theory [20]. Based on these themes, JS developed a codebook. The research team (JS, MG, and LCH) then met and discussed the codebook, making changes to definitions and adding additional codes deductively to ensure that all themes were appropriately represented. The final codebook contained 8 parent codes (over-arching themes) and 24 child codes (each of which corresponded to a more general parent code), and all codes were classified as “I” for ‘inductive’ or “D” for ‘deductive’ so the origin of each code was clear. We (JS and MG) coded all transcripts using the pre-defined codebook so that every interview was double-coded using Dedoose. The team met regularly to discuss coding issues as they came up and to better define codes that contained ambiguity. The two coders compared the codes they had assigned, and when coding differed, they discussed until they reached consensus, consulting LCH as an arbiter. The kappa statistic, calculated based on the 8 parent codes, was 0.75.

### Aim 2: co-design workshop for intervention development

The co-design workshop was conducted at CCPH in June 2019. The eligibility criteria for the co-design workshop remained the same as for the interviews. We used co-design methods to design an intervention encouraging social cohesion and wellness activities among mothers living in the same neighborhood. Our study was designed to include participants in the process to minimize researcher bias. Co-design is defined as including the target audience in the intervention as “constructive participants in the design process of the intervention.” [21]

#### Recruitment

We distributed study fliers at roughly 20 locally based businesses and recruited participants by handing out flyers in playgrounds in the catchment area. Those who resided in the area and were interested in learning about the co-design workshop provided their phone number and we provided additional details and reminders about the co-design workshop via a text or phone call following the initial contact.

#### Co-design workshop

The co-design workshop began with an opening session in which participants introduced themselves, followed by an icebreaker activity encouraging participants to move around the room and meet one another. Children who attended the workshop spent the afternoon in an adjacent room supervised by professional childcare. The themes that emerged from the interviews in Aim 1 informed the structure of the co-design workshop, which comprised 4 stations exploring discussions about (1) technology literacy, (2) free time, (3) physical activity and (4) community champion (see Table 1). Participants were divided into four small groups (maximum 4 participants per group) and were each assigned a workshop station. Each group spent 20 min at each station before rotating to the next so that each group visited 3 of the 4 stations within 1 hour. The discussions at each station were facilitated by a member of the research team and were audio recorded. Each station utilized innovative co-design methods to ensure the thoughts and ideas of each participant were captured (Table 1). The tech literacy station demonstrated the Nextdoor app and participants discussed ways it could be used to organize group activities. The free time station used a poster with two columns for participants to define as a group, the barriers and solutions to creating free time in participant schedules. The physical activity station involved the rotation of a cardboard cube around the group during the discussion to allow participants to write their definitions of physical activity. In the Community Champion station participants wrote their responses to two prompts: 1) What do you want the champion to do? 2) What characteristics are you looking for?

**Table 1:** Co-design workshop stations and methodology

Station	Research Questions to be Answered	Activities	Example questions & probes
1) Tech Literacy	What level(s) of tech literacy are found within the participant group and what barriers are there to using technology to create more free time in their schedules?	Demo: Signing up & basic actions in NextDoor, collect feedback & potential concerns. What is missing?	If intimidated by technology, what can improve or change that? Who are participants comfortable asking for help when they need it (in relation to technology)?
	How we can leverage technology to organize communities for PA/ wellness and related barriers?	Talking about the apps participants use and what they like or dislike about them	If not NextDoor, what would you use to coordinate your schedule with other people to make more time to do things for yourself?
2) Free Time	Define as a group, the barriers to creating free time in participant schedules.	Poster: group discusses barriers to freeing time in one column and potential solutions in the other with room to fill in more as a group	If someone offers to do an activity you’re interested in doing and they say “whatever time works for you”, what time would you set it for? Why is that the ideal time?
	Identify potential solutions to specific scheduling and logistics challenges.		What would make you comfortable leaving your kids with another person?
3) Physical Activity	How does “official” recommendation for exercise as a cancer prevention modifiable behavior fit into participants’ lives?	Looking at a chart of different physical activity options and using post-it notes to “vote” on which ones participants would like to do if they had time	Where is the most convenient place for physical activity to take place? (Are you more likely to go if it’s located--- close to your house or child’s school, etc.)?
	What do participants define as physical activity vs. what health community recommends?	A cardboard cube is passed around the group to facilitate and encourage feedback from all participants. Participants are encouraged to either write their thoughts directly onto the cube or say them out loud and the facilitator acts as a scribe where necessary.	CDC recommends 150 min of of exercise, − how realistic is that for you? How long (maximum) would you typically exercise for if you could? Would you like to do this together with other participants and/or with your children, or alone?
4) Community Champion	Who would participants ‘elect’ to be the person that would encourage and facilitate physical activity for them?	Two themes - 2 large post its, people put post it notes with adjectives / ideas on each one First Post It: What do you want champion to do?	Do you have anyone in your community you look to as a leader? What about that person qualifies them for that role?
	Do participants want the person to model what they’re supposed to be doing OR just someone from their network that they can connect with ?	Second Post It: What characteristics are you looking for?	How would you like your champion to communicate with you?

#### Analysis

The research team met immediately after the conclusion of the workshop to synthesize the results of the co-design workshop, and to complete the intervention design. The team reviewed and discussed all of the recorded participant feedback from each station and recorded the most frequent discussion points or themes that station facilitators heard in discussions with the participants. With the key findings from each station established, the research team then began defining the intervention characteristics, specifically the ‘who,’ ‘what,’ ‘when,’ ‘where’ and ‘how’ of the intervention. They developed a list of potential partner organizations (many of which were named by the participants in the workshop) that would assist with the execution of each aspect of the intervention and discussed the research design for testing the feasibility of the intervention.

## Results

### AIM 1: barriers and facilitators of free time for physical activity

We interviewed 10 women (See Supplemental Table 1 for sample characteristics) and the duration of each interview ranged from 26 to 86 min. At the conclusion of the interviews, many participants appreciated the time to talk about themselves and expressed they seldom had the opportunity to do so. There were 6 major themes that emerged from the interviews. We provide a brief summary below, and detailed quotes from the interviews in Table 2.

**Table 2:** Barriers and facilitators to free time for physical activity based on transcribed semi-structured interviews

Theme	Supporting Quotes
**Limited Time**	
Participant 9	“So I cleaned the kitchen, pack up the lunch, you know for my husband, and put the other food away, wash the dishes, sweep, mop, you know, the regular you have to do the kitchen and pick up all the kids’ stuff … set up the bed for them, check what the homework was about. If he finished. So it looked like [son] didn’t finish some of the homework that he has so I had to go over with him a few things and iron the clothes...You know though sometimes you have got to spend time and talk to them how is school and they want to talk about what happened so I do that with them and then I go into my room and my husband is up and he wants to talk about our weekend...My husband says he is sick then I go to the kitchen bring him some medicine and he is talking more and I don’t remember what happened because he said I fell out. I was gone. He said ‘I was talking to you and you fell asleep’.”
Participant 3	“Everything is for everybody [else], not for me.”
Participant 6	“You don’t feel like concretely anything gets done because it’s constant.”
	“So, once it gets like it feels a little overwhelming to a point where I just kind of like, I’m Iike okay I can’t and I just bag it up and throw and send it to a laundry man so they can do it like if it becomes too much but I try to do it by myself and do it as much as I can so it’s like slowly throughout the week, it’s like taking away time from other stuff because I’m mostly doing laundry.”
Participant 11	“At the end of the day... I am completely exhausted and I am like can I sit down? Can I sit down for half an hour? I just sit down and I am like almost passing out and I am like okay then after that I get up and get the kids you know, brush their teeth or whatever and I am like let me survive.”
**Limited Support**	
Participant 11	“this new bed bug issue is killing me because also it is going to trigger the schizophrenia of my husband and he goes berserk and he starts giving me problems like I don’t need grief from him. It is just like one thing after another.”
Participant 6	“[My husband is] always working, and we don’t have time to sit down...I would like to sit down with my husband … can’t we just have like a breakfast at home twice a week? You know, sit down and the kids, we can actually talk. We have never talked in all these years because he comes home late so I can’t talk to him unless on the phone.
**Physical Activity**	
Participant 3	“I need to get back to my gym because like a year ago I was going to the gym and I felt so great when you go to the gym...it’s like you get all the energy back and it feels so good.“
Participant 10	“[Exercising is] very important for your health like, at this moment I feel like you know I may look young...but you know when you get older that’s when your body needs them, the more you exercise the more strength and the stronger you become. So, it’s important for everyone to start when they are young.”
Participant 9	“I will say maybe if there will be more help with homework with the kids and more help in the house like if there will be that second person that will take care of the cooking part and homework part...I take care of everything else and when I finish doing what I have to do then I put the time in [at the gym].”
Participant 12	“We dance, we go to the park”
Participant 11	“I run after my kids and I mean like I carry groceries.”
Participant 10	“I’m always moving in and around and like I don’t sit down all the time...I’m always doing something.”
Participant 9	“More physical activity than I do? I don’t sit...this is the moment I am sitting down. This is my moment.”
**Self-Efficacy**	
Participant 9	“I schedule my days...when I am home sometimes like I will do certain things like if I finish at night time and I be like let me do some sit-ups. I do some sit-ups.”
Participant 6	“… it’s been so taxing on me and my house that I just suddenly get burned out almost. You can’t do anything...you’re like so tired like always something happening.”
**Trust and Technology**	
Participant 10	“To be honest I don’t know If I would need my kids with another mom especially me not knowing that person.”
Participant 1	“Yes, I think the use of an app is helpful because that’s what everybody goes to...It’s like an app is the easiest way to get people at least to connect … I would definitely use that in that way because of connections.”
Participant 5	“There is also a lot of people that I know that don’t have people around and it does, it does give you some type of comfort to have somebody to share with you something even though where there is the struggle or just to vent so that’s awesome.”
**Community Space**	
Participant 11	Another participant envisioned a center participants could, “just bring their kids for free play and the participants could sit and like drink tea or coffee in one half of the room.”

#### Limited time

We went into the interviews with the assumption that participants did not have much free time for themselves and the interviews confirmed this. All participants but one reported doing significantly more things for their family than for themselves. They frequently described an endless to-do list of chores and caring tasks for their children and partners that left them feeling exhausted. One woman described her day from the moment she got home from work and all tasks revolved around cooking, cleaning, doing homework, and providing emotional support to family members (Participant 9). Another (Participant 3) added, “Everything is for everybody [else], not for me.” One of the constant burdens for many participants was doing laundry. In this urban setting, it was common for laundry facilities to be outside of the apartment either in the basement of the building or at an independent laundromat. For many participants, laundry took up a significant amount of time and was a physical reminder of having too much to do (Participant 1). The running list of things to do for everyone else led participants to feel exhausted with little energy or time for themselves. As Participant 11 said, “At the end of the day... I am completely exhausted and I am like, ‘Can I sit down? Can I sit down for half an hour?’...”

#### Limited support

In addition to having too much work, not enough time and doing many things for others, it was apparent that many participants did not have support from their domestic partners. Nearly every participant had little to no help with household duties or with childcare from their partners. Few participants directly addressed the lack of support, but this was rather conveyed through the absence of partners being mentioned in their descriptions of daily life and workloads. One participant performed all of the caregiving during the week while her husband was at work, and on weekends she described her husband sleeping until the late afternoon. For other participants, their partners were not living in the same house due to incarceration or housing instability. One participant spoke about how her husband’s schizophrenia contributed to her other burdens (Participant 11). Other participants stated that they missed having time with and emotional support from their partner (Participant 6).

#### Physical activity

Participants viewed physical activity as a priority, in addition to mental health and annual clinical examinations. One participant spoke about how good it made her feel in the past when she went to the gym (Participant 3). Another (Participant 10) recognized the importance of exercise for preventative health. Time was mentioned as the main barrier to being able to exercise. Many participants said they could do more physical activity if they had more help at home which would then free up time (Participant 9).

When asked about what participants currently do for physical activity, several defined physical activity as much more than going to the gym or going for runs: “we dance, we go to the park” (Participant 12); “I run after my kids and I mean like I carry groceries” (Participant 11); “I’m always moving in and around and like I don’t sit down all the time...I’m always doing something” (Participant 10). One participant didn’t seem to understand the idea of being able to do more physical activity, stating, “More physical activity than I do? I don’t sit...this is the moment I am sitting down. This is my moment.” (Participant 9).

#### Self-efficacy

Participants had varying levels of self-efficacy. Some displayed high levels of self-efficacy and seemed confident in their abilities to overcome challenges and make positive changes in their lives including exercise. Being able to schedule one’s day was an example of feeling in control, which led to energy and time to “do some sit-ups” (Participant 9). Others seemed overwhelmed and helpless in their own lives, and thus had less control, saying that they “just suddenly get burned out” (Participant 6).

#### Trust and technology

Participants had mixed responses to the idea of using technology to connect with participants in their neighborhood. The idea of not trusting a stranger with one’s children came up many times, whereas, others liked the convenience of an app to connect with others in their neighborhood and acknowledged it “is the easiest way to get people at least to connect” (Participant 1). Participants viewed the app as a way to build community and support, stating, “It does give you some type of comfort to have somebody to share with you something even though where there is the struggle or just to vent so that’s awesome.” (Participant 5).

#### Community space

We closed the interview by asking participants what they would create for themselves if money were no issue. The main thing participants said repeatedly was that they wanted a community center. One participant who grew up in the neighborhood, said that they used to exist, but they had all shut down. The participants distinguished the community center as different from afterschool programs in that it would be a resource for children and mothers like them. Some participants envisioned a place where separate wellness activities for children and mothers occurred simultaneously. Another participant envisioned a center participants could, “just bring their kids for free play and the participants could sit and like drink tea or coffee in one half of the room” (Participant 11).

### Aim 2: co-design workshop

A total of 16 participants attended the co-design workshop at CCPH for 2 hours on a Saturday morning; the group included seven mothers who contributed to the interviews in Aim 1 and the remaining nine were newly recruited for the purpose of the co-design workshop only. The 16 participants were accompanied by a total of 20 children who were cared for by a professional childcare service.

Five key themes emerged from the 4 stations during the workshop, these themes included: (1) preferences for intervention activities; (2) scheduling of intervention activities; (3) facilitation of the intervention; (4) communication and (5) childcare.

#### Preferences for intervention activities

One workstation involved discussions regarding the types of physical activity our target population would like to see implemented. The most frequent requests were for physical activity which included a social aspect, such as group classes, as opposed to being active alone. Participants valued the importance of meeting with other participants in the same neighborhood to share experiences (such as that which took place during the co-design workshop). They also preferred group classes which could accommodate a range of physical fitness levels such as yoga and dance. Walking was also often mentioned as an activity due to its flexibility and low cost. Although the workshop was focused on creating free time to encourage physical activity, participants were interested in education around healthy, affordable eating and cooking.

Participants requested guidance on how and where they could access healthy, affordable food and were seeking advice regarding the guidelines to accessing food pantries in their local area. For example, participants were interested in understanding the income cutoff for qualifying access to food pantries. As with participants’ request for physical activity classes that included a social aspect, they also requested peer support during visits to food pantries in order to reduce the negative stigma associated with visiting these establishments.

Participants were also keen to share and utilize each other’s skills to increase the well-being of their families. For example, one participant offered her skills as an English tutor whilst others offered to teach dance. Participants were very welcoming to the idea of providing a service to the groups in exchange for another service.

#### Scheduling of intervention activities

When asked to describe the features of a feasible intervention, the majority of participants expressed a need for consistency and convenience. They requested the intervention to be delivered in a location that was within their neighborhood and easy to access via foot or a short subway trip. Many participants raised concerns about travelling long distances on the subway with young children. Participants also requested that the intervention was consistently delivered at the same location, time and day of the week.

Whilst the ideal time of day during which the intervention should be delivered varied for participants, it was clear that most would engage in a program that was delivered on a weekend. The workshop reinforced the challenge of delivering a community-based program at a specific time of day that would suit the individual needs of each participant and their families.

#### Facilitation of the intervention

Participants discussed the feasibility of identifying a community champion, a member of their community to facilitate the delivery and co-ordination of the intervention. Participants were encouraged to discuss important leadership characteristics that would encourage them to adhere to the proposed intervention. Participants reported the need for a facilitator who was approachable, kind, friendly and enthusiastic and rated these personal traits with more importance than someone who had ‘expert’ knowledge in delivering exercise classes. Ultimately, participants envisaged a facilitator as someone who would inspire and motivate them to engage in the intervention.

#### Communication

The communication channels used to encourage social cohesion and communication between participants during the intervention was a key element of the workshop. The range in technology literacy varied in our sample. Although some participants were accustomed to downloading and using mobile applications, others were unsure of how to access or download new apps on their devices. None of the participants had used the NextDoor app prior to attending the co-design workshop. We asked workshop attendees to indicate their preference for communication by either NextDoor or text message, or to indicate if they did not have a specific preference for either method. Four participants requested to be contacted via NextDoor, a further four requested text and seven participants indicated no preference.

#### Childcare

All of the participants expressed their appreciation for the professional childcare provided during the workshop to enable their participation. Although the researchers initially envisaged that participants could support one another with childcare duties to enable others to participate in the intervention, the workshop revealed that the majority of participants were not willing to leave their children with other mothers. Some participants liked the idea of taking part in the intervention activities with their children and partners whilst others had a strong preference for having time away from their families to ‘clear their heads’ or have ‘me time.’

#### The FT4W intervention

The co-design workshop resulted in an intervention consisting of 3 months’ worth of sample programming with one activity per week rotating between dance, yoga, food pantry visits and group playdates. With the exception of the food pantry visits, which would be ‘participants only,’ participants would be invited to bring their children to a space that would accommodate one room for the ‘participants only’ activity and a second room in which professional childcare providers would supervise the children. The research team identified Saturday mornings as the ideal timeframe. Because participants expressed different communication preferences during the workshop, we decided half the group would be assigned to receive communication from the community champion via text message, and the other half would receive communication via NextDoor messages. We also determined ‘ground rules’ for communication that would be distributed to participants. Collectively, by participating in the group activities and communicating through neighborhood-based apps or text messaging, the intervention was designed to increase social cohesion that would then feedback into greater participation. The protocol to test the feasibility of this intervention is described elsewhere [17].

## Discussion

Through this two-phased co-design process, we created an intervention with mothers in the Washington Heights community with the goal of using technology to bring mothers together for wellness. By interviewing participants one-on-one, we confirmed they had little time and limited outside support which hindered them from increasing their own physical activity; we also learned that in addition to physical health, mental health was a concern for participants. Our interviews also highlighted that participants in the community had varying degrees of self-efficacy and trust in technology. By bringing mothers and researchers together in a co-design workshop, we discovered the types of physical activities they would enjoy participating in, the ideal time to do so, the kind of childcare they needed, and how they wished to communicate with study staff, the community champions and each other. Both phases highlighted the need for a community space that mothers and children could co-occupy.

There were important components of the intervention that came directly from participants. Addressing food insecurity was one need that we had not anticipated. Whilst our initial objective was to use technology to promote physical activity to enhance cancer prevention [22], the co-design process revealed that our target group wanted an all-encompassing approach that included mental health and access to healthy foods as well as physical activity. Given the importance of nutrition in obesity prevention, this was an important discovery that bodes well for a more comprehensive intervention with substantial potential for prevention of cancer and other chronic diseases. As an early expansion upon our original idea, we were able to include exclusive access to a food pantry as one of the four group activities. The potential benefits of this activity were two-fold, in that it could increase social cohesion through a group volunteer activity as well as provide access to free healthy, fresh produce and food for those individuals in need. Other studies of cancer prevention aimed at reducing obesity have incorporated both physical activity and healthy eating [23, 24] but few have incorporated mechanisms to reduce the social and economic barriers to accessing ways to effect change in both drivers [5, 24]. Given the current pandemic, this may be even more important for effective implementation.

Mothers greatly appreciated the set-up of the workshop itself, namely having their children cared for in a nearby room at the same time giving them space to connect with other mothers. Although we originally envisioned having mothers share the childcare load themselves, participants did not feel comfortable having other participants care for their children. It was clear that the participants greatly valued access to professional childcare and that this should be included in the intervention. Other studies have identified childcare as a barrier to engagement in physical activity in mothers, but few interventions have provided childcare to actually enable mothers' participation. [5].

We also adapted our use of technology to communicate with participants based on their feedback. The initial study design intended for all participants to receive communication and reminders about the intervention via NextDoor. However, we learned from the co-design workshop that not all participants were comfortable with or likely to use a new or unfamiliar app. Therefore, we designed the intervention so that participants could communicate via either communication channel: NextDoor or text message (SMS), depending on their preferences. We plan to create a curriculum, so that the same messages are sent regardless of channel. Thus, ‘communication assignment’ is a variable through which we can compare and evaluate the efficacy of two different channels of communication for this type of intervention. Text messaging is a proven form of communication in interventions studies [5, 9, 25] and investigating whether the more sophisticated technological affordances that most apps offer (compared to text messages) improve intervention (fidelity, feasibility, efficacy, effectiveness, outcomes) is a question worth further research.

The non-random selection of a small number of participants and inclusion of only English-speakers may have biased our sample. However, researchers in Australia, completely independent from our team, have designed a similar intervention that shares many of the same features including community champion-type role (regular text communication including encouragement, reminders), recommending activities with childcare and self-reported physical activity behaviors [5]. Such parallels from different populations in starkly different contexts suggests that there may be some universal applications of our findings and co-design intervention to other settings.

Our next step is to assess the feasibility of the intervention [17]. We plan to roll out the intervention in Washington Heights with the same participants, if willing, and administer baseline and follow-up questionnaires. At baseline and follow-up we will measure social cohesion, physical activity, health status, and well-being. At follow-up, we will re-assess these measures and additionally collect participation rates and feedback regarding time and scheduling, acceptability, and cost effectiveness of the intervention [26]. Collectively these measures will give us an overall assessment of feasibility. In addition to feasibility, there is ample opportunity within the field of dissemination and implementation science to investigate how the Free Time for Wellness intervention can be scaled and adapted through hybrid study-designs that evaluate both efficacy and effectiveness simultaneously, while also taking into account cost-effectiveness.

## Conclusions

Through this two-phased co-design process, we created an intervention with mothers in an urban community with the goal of using social media to bring mothers together for wellness primarily through increased physical activity. Our locally-based approach with a specific community helped tailor the intervention to their needs, however, there are some universal applications of our findings, and of the co-design workshop model, to other settings and studies interested in administering community-based health interventions.

## Supplementary Information

**Additional file 1: Supplemental Table 1.** Participant Characteristics.

**Additional file 2:.** Interview Guide.

## Data Availability

The datasets generated and/or analyzed during the current study are not publicly available due to efforts to protect the privacy of individuals, but deidentified data are available from the corresponding author on reasonable request.

## Abbreviations

FT4W: Free Time for Wellness
CRUK: Cancer Research United Kingdom
CCPH: Columbia Community Partnership for Health
CECR: Community Engagement Core Resource
