# Supplementary material for: Free Time For Wellness: a co-designed intervention utilizing social networks to encourage physical activity for cancer prevention among low resourced mothers
Source: BMC Public Health. 2021 Oct 7;21:1805. doi: 10.1186/s12889-021-11775-9 (PMC8499394; doi:10.1186/s12889-021-11775-9)
Supplement: Supplementary file 1 — Additional file 1: Supplemental Table 1. Participant Characteristics. [file 12889_2021_11775_MOESM1_ESM.docx]

Supplemental Table 1: Participant Characteristics

| **Age** | **Ethnicity** | **Number of children** | **Ages of children** | **FT4W Participation** |
| --- | --- | --- | --- | --- |
| 38 | Latinx | 2 | 4, 11 | Interview |
| 36 | Latinx | 3 | 5, 10, 16 | Interview |
| 36 | LatinX | 1 | 11 | Interview |
| 46 | Black | 3 | 3, 10, 12 | Interview |
| 37 | LatinX | 2 | 6, 15 | Interview, workshop |
| 40 | Asian | 5 | 3, 4, 7, 16, 18 | Interview, workshop |
| 38 | LatinX | 2 | 5, 19 | Interview, workshop |
| 42 | LatinX | 3 | 4, 7, 8 | Interview, workshop, intervention |
| 45 | White | 2 | 4, 6 | Interview, workshop, intervention |
| 32 | Asian | 1 | 4 | Interview, workshop, intervention |
| Not collected | Not collected | Not collected | Not collected | workshop |
| Not collected | Not collected | Not collected | Not collected | workshop |
| Not collected | Not collected | Not collected | Not collected | workshop |
| Not collected | Not collected | Not collected | Not collected | workshop |
| Not collected | Not collected | Not collected | Not collected | workshop |
| 32 | LatinX | 2 | 5,3 | workshop, intervention |
| 34 | Asian | 1 | 4 | workshop, intervention |
| 32 | White | 1 | 3 | workshop, intervention |
| 47 | White | 3 | 7, 5, 5 | workshop, intervention |
| 24 | LatinX | 1 | 3 | workshop, intervention |
