# Supplementary material for: Free Time For Wellness: a co-designed intervention utilizing social networks to encourage physical activity for cancer prevention among low resourced mothers
Source: BMC Public Health. 2021 Oct 7;21:1805. doi: 10.1186/s12889-021-11775-9 (PMC8499394; doi:10.1186/s12889-021-11775-9)
Supplement: Supplementary file 2 — Additional file 2:. Interview Guide. [file 12889_2021_11775_MOESM2_ESM.docx]

Interview Guide

Time

- *Exploring the types of activities mothers currently do or might like to do /Exploring mothers’ attitudes, preferences and motivations about increasing free time for themselves and their families*
  - Tell me about your day yesterday.
  - Tell me about Saturday.
  - Do you have a to do list? Where is it kept? In your mind, on paper, on your phone? What is on it now?
    - Do you have enough time to do the items on that list?
    - If you had more time, what would you do with it?
    - Of those item on your list how many are for yourself?
    - Do you have enough time to do the things you want to do for yourself?
  - If you don’t have a to do list, tell me how you manage your time and responsibilities.
  - Could you please draw us a journey map of this past Wednesday, starting from when you woke up until when you went to bed? Above the line represents high moments in your day, and below the line represents low moments. As you go, please tell us what made those moments good or bad.

Health

- *Understanding the barriers and facilitators affecting PA in families/Understanding the context and culture of PA in families*
  - In general, would you say that your health is:
    - Excellent
    - very good
    - good
    - fair
    - poor
  - What aspects of your health are important to you?
  - What do you do for physical activity?
  - What do other members in your family do for physical activity?
  - What things keep you and/or your family from physical activity?
  - Did your parents do physical activity when you grew up?
- *Exploring mothers’ attitudes, preferences and motivations about increasing PA for themselves and their families’/Exploring factors to increase adherence to a sustainable PA routine*
  - What do you currently do for physical activity?
  - What do and your family members currently do for physical activity?
  - Do you think you and/or members of your family should do more physical activity? Why or why not?
  - How would you encourage them to do more physical activity?
  - What would it take for you to do more physical activity?
  - What type of physical activity would you do?
  - Would you do it alone or with others? Who would you do it with?
  - Have you tried increasing your physical activity before?
  - How long did it last?
  - What worked, what didn’t work?

Smartphones

- *App use (we should make eligibility criteria that they are smartphone users)/ Understanding their attitudes and preferences about using Nextdoor to (1) connect with other mothers in their neighbourhood, (2) be physically active*
  - What Apps do you use the most?
  - How do you communicate with your family and friends?
  - How do you communicate with them?
  - Do you have any physical activity Apps? How often do you use them?
  - Do you track how much time you spend on your phone? Can we look at your phone to see how much time you spend on each app?
  - Do you use an app called Nextdoor?
- We have an idea to use NextDoor to connect moms in neighborhoods to each other so that they can connect and create free time to be healthy together. We are thinking moms could exercise together or exchange childcare so that one mom could exercise while the other takes care of her children.
  - Is this something you would use? Why/why not?
  - Tell us your honest thoughts about this idea?

Resources

- Are you friends with other mothers in your neighborhood?
- How many people do you have near you that you can count on for real help in times of trouble or difficulty, such as watching over children or pets, giving rides to the hospital or store, or helping if you are sick? [Response categories: 0, 1, 2-5, 6-9, 10 or more].
- During the past month, have your financial resources been adequate to meet your daily needs? [Response categories: yes, no]
